# Recreational physical activity and ovarian cancer risk in African American women

**DOI:** 10.1002/cam4.677

**Published:** 2016-02-29

**Authors:** Sarah E. Abbott, Elisa V. Bandera, Bo Qin, Lauren C. Peres, Patricia G. Moorman, Jill Barnholtz‐Sloan, Ann G. Schwartz, Ellen Funkhouser, Edward S. Peters, Michele L. Cote, Anthony J. Alberg, Paul Terry, Melissa Bondy, Lisa E. Paddock, Sydnee Crankshaw, Frances Wang, Fabian Camacho, Joellen M. Schildkraut

**Affiliations:** ^1^ Department of Public Health SciencesUniversity of VirginiaCharlottesvilleVirginia; ^2^ Department of Population ScienceRutgers Cancer Institute of New JerseyNew BrunswickNew Jersey; ^3^ Department of Community and Family MedicineDuke University Medical CenterDurhamNorth Carolina; ^4^ Case Comprehensive Cancer CenterCase Western Reserve University School of MedicineClevelandOhio; ^5^ Department of Oncology and the Karmanos Cancer Institute Population Studies and Disparities Research ProgramWayne State UniversityDetroitMichigan; ^6^ Division of Preventive MedicineUniversity of Alabama at BirminghamBirminghamAlabama; ^7^ Epidemiology ProgramLouisiana State University Health Sciences Center School of Public HealthNew OrleansLos Angeles; ^8^ Hollings Cancer Center and Department of Public Health SciencesMedical University of South CarolinaCharlestonSouth Carolina; ^9^ Department of MedicineUniversity of Tennessee Medical Center‐KnoxvilleKnoxvilleTennessee; ^10^ Cancer Prevention and Population Sciences ProgramBaylor College of MedicineHoustonTexas; ^11^ Cancer Research ProgramNew Jersey State Cancer RegistryRutgers Cancer Institute of New JerseyNew BrunswickNew Jersey; ^12^ Duke Cancer InstituteDuke University Medical CenterDurhamNorth Carolina

**Keywords:** African American, ovarian cancer, physical activity

## Abstract

The literature on recreational physical activity (RPA) and ovarian cancer risk is inconclusive and most studies of RPA and ovarian cancer have been conducted in white populations. This study is the first to investigate the association between RPA and ovarian cancer in an exclusively African American (AA) population. We analyzed data from an ongoing U.S. population‐based, case–control study of AA women, which included 393 women recently diagnosed with invasive epithelial ovarian cancer (IEOC) and 611 controls. A baseline interview assessed RPA frequency, intensity, and duration. Each RPA intensity was assigned a metabolic equivalent of task (MET) value and MET‐min/week were calculated. Unconditional multivariable logistic regression was performed to investigate associations between RPA and IEOC risk. Compared with sedentary women, predominantly mild intensity RPA was significantly inversely associated with IEOC risk for women reporting above median (>297) MET‐min/week (odds ratio [OR] = 0.52; 95% confidence interval [CI]: 0.34, 0.78) and nonsignificantly for <297 MET‐min/week (OR = 0.71; 95% CI: 0.44, 1.12). Predominantly moderate intensity RPA was associated with significantly increased risk for women reporting above median (>540) MET‐min/week (OR = 1.51; 95% CI: 1.03, 2.23). Predominantly strenuous intensity RPA was nonsignificantly associated with lower IEOC risk for women reporting above median (>1800) MET‐min/week (OR = 0.72; 95% CI: 0.33, 1.57). The inverse associations for mild and strenuous intensity RPA were most pronounced in obese women (body mass index >30 kg/m^2^). The findings that mild and strenuous RPA may reduce the risk of IEOC particularly among obese women are difficult to reconcile with the increased risk observed for moderate RPA. Further research is warranted to determine whether these findings are genuine and, if so, their mechanistic basis.

## Introduction

An inverse association between physical activity and breast, colon, and endometrial cancers has been consistently observed in epidemiologic studies 1, 2. The literature on the role of recreational physical activity (RPA) in relation to ovarian cancer risk, however, is inconclusive 2. Seventeen studies have reported inverse associations for total RPA 3, 4, 5, 6, 7, 8, 9, 10, 11, 12, 13, 14, 15, 16, 17, 18, 19, although only six observed statistically significant associations 3, 8, 9, 10, 13, 17, while five have reported positive associations, one statistically significant 20 and the remainder nonsignificant 21, 22, 23, 24. The results have remained inconsistent even when RPA intensity has specifically been investigated. Several studies have reported increased risk associated with vigorous intensity RPA 6, 19, 21, whereas others observed decreased risk 5, 8, 17, 25. Most studies 3, 5, 6, 7, 8, 10, 14, 16, 19, 23, 25 have reported decreased risk associated with moderate intensity RPA, but a few studies 20, 21 have reported increased risk.

This remains a topic of considerable importance as ovarian cancer survival rates are poor 26 and the role of modifiable lifestyle factors such as physical activity in ovarian cancer prevention is key but is not well understood. At least some of the inconsistency in the evidence may be overcome by improving measurement of physical activity to include the frequency, intensity, and duration of physical activity. More refined measurement of physical activity could lead to new insights as many of the prior studies of this topic have not collected information on all three domains of physical activity 2. Additionally, many studies have used referent groups that included women reporting low amounts of RPA instead of restricting the referent to sedentary women 2; this would bias results toward the null. Furthermore, the majority of studies on physical activity and ovarian cancer have been conducted in white populations, although African Americans (AA) exhibit lower levels of physical activity 27 and worse 5‐year ovarian cancer survival 28. The present study uses data from a large population‐based case–control study of invasive epithelial ovarian cancer (IEOC) in AA women to examine associations between RPA and risk of IEOC for the first time in an exclusively AA population.

## Methods

### Study population

The African American Cancer Epidemiology Study (AACES) is an ongoing, population‐based case–control study of IEOC in AA women in 11 geographic locations (Alabama, Georgia, Illinois, Louisiana, Michigan, New Jersey, North Carolina, Ohio, South Carolina, Tennessee, and Texas). Institutional review board approval was obtained from Duke University (the lead institution for the study) and all participating institutions, and informed consent was obtained from all individual participants included in the study. Methods have been described in detail elsewhere 29. Briefly, eligible cases include AA women between 20 and 79 years of age with newly diagnosed IEOC. Controls are AA women with at least one intact ovary and no history of ovarian cancer, frequency matched to cases on state of residence and 5‐year age categories. Participants completed a baseline computer‐assisted telephone interview, administered by a trained interviewer, which includes detailed questions on demographic characteristics; reproductive, gynecologic and medical history; exogenous hormone use including hormone therapy (HT) and oral contraceptives (OC); family history; and lifestyle characteristics including smoking, alcohol consumption, body size, and physical activity. Food frequency questionnaires, annual follow‐up surveys, biospecimens, and medical records are also obtained. Recruitment began in December 2010; as of January 2015, 469 cases and 705 controls completed the full questionnaire. Eligibility was restricted to participants for whom physical activity data and all covariates were available, resulting in a final sample size of 393 cases and 611 controls.

### Recreational physical activity

In the baseline interview, participants were asked to self‐report the usual amount of RPA they engaged in each week. Participants were asked to recall their average weekly physical activity 1 year prior to their diagnosis (cases) or during the past year (controls). They were instructed to include only exercise sessions that lasted 10 min or longer which were completed during free time, excluding occupational activity and housework. The interview questions (provided in Table 1), adapted from the International Physical Activity Questionnaire 30, asked about strenuous, moderate, and mild intensity exercise, and included examples of each type of activity. Participants reported the times per week they engaged in each type of exercise and the average length of each exercise session. This information was used to calculate total weekly minutes of RPA for each intensity level.

**Table 1:** Physical activity questions included in the African American Cancer Epidemiology Study questionnaire

Considering a typical week, how many times on average did you perform the following kinds of exercise?	Examples
Strenuous‐intensity exercise where your heart beats rapidly and you sweat	Running, aerobics class, cross‐country skiing, vigorous swimming, vigorous bicycling
Moderate‐intensity exercise which is not exhausting and you may perspire lightly	Fast walking, tennis, easy bicycling, easy swimming, dancing
Mild‐intensity exercise which requires minimal effort and does not make you sweat	Easy walking, yoga, bowling, shuffleboard, golf

Each intensity level was assigned a metabolic equivalent of task (MET) score according to the International Physical Activity Questionnaire guidelines 30. METs are a method of expressing the energy costs of different activities as a multiple of resting metabolic rate 31. MET‐min per week 32 were calculated for each participant using the following equation:

$$\begin{array}{cc} & \text{MET‐min/week}=3.3\;\text{METs}{\;}^{*}\;\left(\text{min/week mild intensity RPA}\right)\\ & \;+\;4.0\;{\text{METs}}^{*}\left(\text{min/week moderate intensity RPA}\right)\\ & \;+\;8.0\;{\text{METs}}^{*}\left(\text{min/week strenuous intensity RPA}\right). \end{array}$$

### Statistical analysis

The prevalence of demographic characteristics was calculated and chi‐square tests (for categorical variables) and *t*‐tests (for continuous variables) were performed to compare the distributions and mean values between cases and controls. Unconditional multivariable logistic regression was employed to estimate the odds ratios (ORs) and 95% confidence intervals (95% CIs) for the association between MET‐min/week of RPA and the risk of ovarian cancer. Sedentary participants (0 MET‐min/week) were used as the referent group and those reporting any regular weekly physical activity were divided into tertiles according to the distribution of MET‐min/week among the controls.

In addition, unconditional multivariable logistic regression was performed to estimate ORs for IEOC risk according to participants' predominant RPA intensity. For this analysis, participants were classified according to the intensity for which they reported the greatest min/week. The majority of participants reported spending at least 50% of their total activity time at one intensity; in the event of a tie between two or more intensities (*n* = 24), participants were classified according to the higher intensity.

Finally, unconditional multivariable logistic regression was performed to estimate ORs for IEOC risk according to MET‐min/week by participants' predominant RPA intensity category, with sedentary participants as the referent for each analysis. For these analyses, participants are categorized according to their predominant RPA intensity but their MET‐min/week reflect all reported intensities, thus adjusting for frequency and duration by intensity. MET‐min were dichotomized based on the median MET‐min/week reported by controls (297 for mild, 540 for moderate, and 1800 for strenuous).

Covariates included in the multivariable analyses as confounders were reference age, defined as age at diagnosis for cases and age at baseline interview for controls (<40, 40–59, >60); study site (Alabama, Louisiana, New Jersey, North Carolina, Ohio, South Carolina, Texas, Michigan and Illinois [combined due to sample size and regional similarities], Georgia, and Tennessee [combined due to sample size and regional similarities]); education (high school or less, some post‐high school training, college or graduate degree); smoking status (never, current, former); parity (0, 1, 2, 3+); duration of OC use (never, <60 months, 60+ months), ever use of HT (yes/no), body mass index (BMI) (<25, 25–29.9, >30 kg/m^2^), history of tubal ligation (yes/no); family history of breast or ovarian cancer in a first‐degree relative (yes/no); occupational physical activity (mainly sitting, mainly standing/walking, mainly active, do not work outside home), total energy intake (kcal/day), and total RPA (min/week). BMI (<30 and >30 kg/m^2^) and menopausal status (pre/postmenopausal) were examined as effect modifiers. A sensitivity analysis of serous only cases was conducted. All analyses were performed using SAS version 9.4 (Cary, NC, USA).

## Results

Table 2 presents demographic and descriptive characteristics of the study participants. On average, compared to controls, cases were statistically significantly older (mean age 57.3 vs. 54.5 years), had lower parity, were more likely to have a first‐degree family history of breast or ovarian cancer and to be former smokers, had fewer months of OC use, and had a higher mean BMI. Cases were also less likely to have had a tubal ligation compared to controls (*P* = 0.06) and less likely to have received post‐high school education (*P* = 0.06). Total energy intake, occupational physical activity level, and menopausal status did not differ significantly between cases and controls.

**Table 2:** Demographic characteristics of African American women with and without invasive epithelial ovarian cancer, African American Cancer Epidemiology Study 2010–January 2015

	Cases*n* = 393*n* (%)	Controls*n* = 611*n* (%)	*P*‐value
Mean age (years) (SD)	57.3 (10.6)	54.5 (11.6)	<0.01
Age (years)			<0.01
<40	18 (4.6)	69 (11.3)	
40–59	212 (53.9)	336 (55.0)	
60+	163 (41.5)	206 (33.7)	
Education			0.06
High school or less	175 (44.5)	226 (37.0)	
Some post‐high school training	103 (26.2)	176 (28.8)	
College or graduate degree	115 (29.3)	209 (34.2)	
Mean body mass index (BMI, kg/m^2^) (SD)	33.3 (8.8)	32.2 (8.1)	0.04
BMI (kg/m^2^)			0.44
<18.5 (underweight)	5 (1.3)	9 (1.5)	
18.5–24.9 (normal weight)	51 (13.0)	102 (16.7)	
25–29.9 (overweight)	104 (26.5)	152 (24.9)	
>30 (obese)	233 (59.3)	348 (57.0)	
Parity (no. of live births)			<0.01
0	81 (20.6)	81 (13.3)	
1	80 (20.4)	108 (17.7)	
2	89 (22.7)	165 (27.0)	
3+	143 (36.4)	257 (42.1)	
Tubal ligation			0.06
Yes	136 (34.6)	247 (40.4)	
No	257 (65.4)	364 (59.6)	
Oral contraceptive use			<0.01
Never	113 (28.8)	122 (20.0)	
<60 months	160 (40.7)	275 (45.0)	
60+ months	120 (30.5)	214 (35.0)	
Ever use of hormone therapy			0.22
Yes	79 (20.1)	104 (17.0)	
No	314 (79.9)	507 (83.0)	
Family history of breast or ovarian cancer (first‐degree relative)			0.02
Yes	96 (24.4)	111 (18.2)	
No	297 (75.6)	500 (81.8)	
Menopausal status^a^			0.21
Premenopausal	108 (27.5)	190 (31.2)	
Postmenopausal	285 (72.5)	419 (68.8)	
Smoking status			<0.01
Never	221 (56.2)	350 (57.3)	
Current	42 (10.7)	121 (19.8)	
Former	130 (33.1)	140 (22.9)	
Occupational physical activity			0.30
Mainly sitting	66 (16.8)	118 (19.3)	
Mainly standing or walking	68 (17.3)	81 (13.3)	
Mainly active	63 (16.0)	95 (15.6)	
Do not work outside the home	196 (49.9)	317 (51.9)	
Mean total energy intake (kcal/day) (SD)	1813 (1487)	1756 (1135)	0.52

a Missing data on menopausal status for two controls.

Compared with sedentary women, the OR for any RPA in relation to IEOC was 0.79 (95% CI: 0.57, 1.11), and the ORs for tertiles of total RPA in relation to IEOC were 0.78 (95% CI: 0.53, 1.14), 0.80 (95% CI: 0.52, 1.21), and 1.00 (95% CI: 0.58, 1.74) for 1 to <320, 320 to <720, and ≥720 MET‐min/week, respectively (Table 3). A significant inverse association was observed for women who engaged in mild intensity RPA only (OR = 0.60; 95% CI: 0.41, 0.88) and a nonsignificant inverse association was observed for women reporting any strenuous RPA (OR = 0.82; 95% CI: 0.41, 1.64), while a nonsignificant, positive association was observed for women reporting any moderate but no strenuous intensity RPA (OR = 1.08; 95% CI: 0.73, 1.60).

**Table 3:** Odds ratios for the association between MET‐min/week and highest reported intensity of RPA and invasive epithelial ovarian cancer, African American Cancer Epidemiology Study 2010–January 2015

	Cases*n* (%)	Controls*n* (%)	OR^a^	95% CI
Any RPA				
Sedentary (0 MET‐min/week)	142 (36.1)	198 (32.4)	1.00	Referent
Any RPA (>0 MET‐min/week)	251 (63.9)	413 (67.6)	0.79	(0.57, 1.11)
Total RPA, MET‐min/week				
Sedentary (0 MET‐min/week)	142 (36.1)	198 (32.4)	1.00	Referent
T1 (<320 MET‐min/week)	77 (19.6)	136 (22.3)	0.78	(0.53, 1.14)
T2 (320 to <720 MET‐min/week)	69 (17.6)	127 (20.8)	0.80	(0.52, 1.22)
T3 (>720 MET‐min/week)	105 (26.7)	150 (24.6)	1.00	(0.58, 1.74)
Predominant RPA intensity				
Sedentary	142 (36.1)	198 (32.4)	1.00	Referent
Mild	79 (20.1)	171 (28.0)	0.60	(0.41, 0.88)
Moderate	147 (37.4)	193 (31.6)	1.08	(0.73, 1.60)
Strenuous	25 (6.4)	49 (8.0)	0.82	(0.41, 1.64)

RPA, recreational physical activity; OR, odds ratio; CI, confidence interval; MET, metabolic equivalent of task.

a Adjusted for age, study site, education, smoking, tubal ligation, parity, oral contraceptive use, hormone therapy use, body mass index, total energy intake, occupational physical activity, family history of breast or ovarian cancer, and total RPA (min/week).

We further analyzed MET‐min/week for above and below the median by participants' predominant RPA intensity (Table 4). Generally, our results do not suggest a dose–response association. Among participants who performed predominantly mild intensity RPA, we observed inverse associations for <297 and >297 MET‐min/week (OR = 0.71; 95% CI: 0.44, 1.12 and OR = 0.52; 95% CI: 0.34, 0.78, respectively) versus sedentary women. Of those reporting predominantly strenuous intensity RPA, a nonsignificant inverse association of similar magnitude was observed for those reporting >1800 MET‐min/week (OR = 0.72; 95% CI: 0.33, 1.57) while the association for <1800 MET‐min/week was positive and nonsignificant (OR = 1.09; 95% CI: 0.49, 2.41). ORs greater than 1 were observed for participants reporting predominantly moderate intensity RPA for <540 MET‐min/week (OR = 1.22; 95% CI: 0.82, 1.81) and for >540 MET‐min/week (OR = 1.51; 95% CI: 1.03, 2.23).

**Table 4:** Odds ratios for the association between MET‐min/week of RPA by intensity and invasive epithelial ovarian cancer, African American Cancer Epidemiology Study 2010–January 2015

MET‐min/week	Cases*n* (%)	Controls*n* (%)	OR^a^	95% CI
Predominant RPA intensity^b^				
Mild				
Sedentary (0 MET‐min/week)	142 (64.3)	198 (53.7)	1.00	Referent
<297 MET‐min/week	35 (15.8)	65 (17.6)	0.71	(0.44, 1.12)
>297 MET‐min/week	44 (19.9)	106 (28.7)	0.52	(0.34, 0.78)
Moderate				
Sedentary (0 MET‐min/week)	142 (49.5)	198 (50.6)	1.00	Referent
<540 MET‐min/week	60 (20.9)	91 (23.3)	1.22	(0.82, 1.81)
>540 MET‐min/week	85 (29.6)	102 (26.1)	1.51	(1.03, 2.23)
Strenuous				
Sedentary (0 MET‐min/week)	142 (85.5)	198 (80.2)	1.00	Referent
<1800 MET‐min/week	11 (6.6)	22 (8.9)	1.09	(0.49, 2.41)
>1800 MET‐min/week	13 (7.8)	27 (10.9)	0.72	(0.33, 1.57)

MET, metabolic equivalent of task; RPA, recreational physical activity; OR, odds ratio; CI, confidence interval.

a Adjusted for age, study site, education, smoking, tubal ligation, parity, oral contraceptives use, hormone therapy use, body mass index, total energy intake, occupational physical activity, family history of breast or ovarian cancer, and total RPA (min/week).

b A separate regression model was used for each intensity.

As BMI is a potential effect modifier for the association between RPA and cancer 2, we also stratified analyses by BMI (<30 and >30 kg/m^2^). Any regular RPA was nonsignificantly inversely associated with IEOC for obese (BMI >30 kg/m^2^) women (OR = 0.79; 95% CI: 0.51, 1.21) but not for women with a BMI <30 kg/m^2^ (OR = 0.99; 95% CI: 0.54, 1.81) (Table 5). Among obese women, those in the lowest tertile of RPA had the strongest inverse association with IEOC (OR = 0.69; 95% CI: 0.42, 1.13), while the second tertile had a weaker inverse association (OR = 0.91; 95% CI: 0.52, 1.58) and the third tertile had a weak positive association (OR = 1.06; 95% CI: 0.52, 2.19). No trend was observed among women with BMI <30 kg/m^2^ (ORs from 0.83 to 1.04, all nonsignificant). Predominantly mild intensity RPA was significantly inversely associated with IEOC among obese women (OR = 0.53; 95% CI: 0.33, 0.87) and nonsignificantly among nonobese women (OR = 0.87; 95% CI: 0.44, 1.75). Predominantly strenuous intensity RPA was nonsignificantly inversely associated with IEOC among obese women (OR = 0.45; 95% CI: 0.14, 1.51), but not nonobese women (OR = 1.04; 95% CI: 0.39, 2.82). ORs for predominantly moderate intensity RPA were >1 for both obese (OR = 1.35; 95% CI: 0.80, 2.27) and nonobese women (OR = 1.10; 95% CI: 0.57, 2.13). Tests for interaction between BMI and RPA were not significant. We conducted analyses stratified by menopausal status but did not observe any substantial differences between pre‐ and postmenopausal women (data not shown). Analyses restricted to serous only cases (*n* = 271) were not substantially different from the analyses of all subtypes (data not shown).

**Table 5:** Odds ratios for the association between MET‐min/week of RPA and invasive epithelial ovarian cancer stratified by body mass index, African American Cancer Epidemiology Study 2010–January 2015

Body mass index (BMI)									
	<30 kg/m^2^				>30 kg/m^2^				
	Cases*n* (%)	Controls*n* (%)	OR^a^	95% CI	Cases*n* (%)	Controls*n* (%)	OR^a^	95% CI	Interaction*P*‐value^b^
Any RPA									
Sedentary (0 MET‐min/week)	46 (28.9)	76 (28.9)	1.00	Referent	96 (41.2)	122 (35.1)	1.00	Referent	
Any RPA (>0 MET‐min/week)	114 (71.3)	187 (71.1)	0.99	(0.54, 1.81)	137 (58.8)	226 (64.9)	0.79	(0.51, 1.21)	0.35
Tertiles of RPA									
Sedentary (0 MET‐min/week)	46 (28.8)	76 (28.9)	1.00	Referent	96 (41.2)	122 (35.1)	1.00	Referent	
T1 (<320 MET‐min/week)	30 (18.8)	56 (21.3)	1.10	(0.57, 2.16)	47 (20.2)	80 (23.0)	0.69	(0.42, 1.13)	0.40
T2 (320 to <720 MET‐min/week)	25 (15.6)	53 (21.2)	0.83	(0.39, 1.77)	44 (18.9)	74 (21.3)	0.91	(0.52, 1.58)	0.35
T3 (>720 MET‐min/week)	59 (36.9)	78 (29.7)	1.04	(0.39, 2.73)	46 (19.7)	72 (20.7)	1.06	(0.52, 2.19)	0.32
Predominant RPA intensity									
Sedentary	46 (28.8)	76 (28.9)	1.00	Referent	96 (41.2)	122 (35.1)	1.00	Referent	
Mild	31 (19.4)	61 (23.2)	0.87	(0.44, 1.75)	48 (20.6)	110 (31.6)	0.53	(0.33, 0.87)	0.18
Moderate	63 (39.4)	96 (36.5)	1.10	(0.57, 2.13)	84 (36.1)	97 (27.9)	1.35	(0.80, 2.27)	0.22
Strenuous	20 (12.5)	30 (11.4)	1.04	(0.39, 2.82)	5 (2.2)	19 (5.5)	0.45	(0.14, 1.51)	0.12

MET, metabolic equivalent of task; RPA, recreational physical activity; OR, odds ratio; CI, confidence interval.

a Adjusted for age, study site, education, smoking, tubal ligation, parity, oral contraceptive use, hormone therapy use, BMI, total energy intake, occupational physical activity, family history of breast or ovarian cancer, and total RPA (min/week).

b Test of interaction *P*‐value based on pooled analysis.

## Discussion

The present study observed a statistically significant inverse association between predominantly mild‐intensity RPA and IEOC as well as a nonsignificant inverse association for predominantly strenuous intensity. Conversely, a positive association was observed between moderate‐intensity RPA and IEOC, which was significant for women reporting above median (>540) MET‐min/week. Subgroup analyses revealed that the inverse associations for mild, strenuous, and total RPA were largely concentrated among women with BMI ≥30 kg/m^2^.

Various biological mechanisms for an association between physical activity and IEOC have been proposed. Physical activity may reduce IEOC risk by reducing BMI; there is evidence of an inverse association between RPA and BMI 2 and studies have observed a direct association between BMI and IEOC risk 2, 33. However, associations between BMI and IEOC risk differ by histologic subtype and a pooled analysis by the Ovarian Cancer Association Consortium did not find an association with high‐grade serous invasive tumors, the most common IEOC subtype 34. Several hormonal mechanisms have also been hypothesized. Vigorous physical activity has been suggested to reduce IEOC risk by suppressing ovulation in premenopausal women 33. Some investigators have suggested that physical activity reduces the risk of IEOC by reducing endogenous estrogen levels 2, 19. Conversely, it has been suggested that gonadotropin production is increased in response to reduced circulating estrogens, increasing IEOC risk 2. Further, Risch 35 has hypothesized that physical activity increases IEOC risk through reduced progesterone levels and increased androgen levels.

Differences in the associations with physical activity have been observed between case–control and prospective studies of physical activity and ovarian cancer, with case–control studies more likely to observe inverse associations and cohort studies more likely to observe null or positive associations 2, 36.

Additional differences in exposure classification across studies make comparison of our results for RPA intensity with prior studies difficult. While AACES distinguished between three levels of intensity, the majority of studies have classified intensity as either moderate or vigorous, with one cohort study additionally assessing nonoccupational walking as a distinct exposure 20. Many studies have classified all walking as moderate intensity, whereas AACES distinguishes between easy walking (mild intensity) and fast walking (moderate intensity).

A recent study of physical activity and IEOC in the Nurses' Health Study and Nurses' Health Study II cohorts by Huang et al. 33 found no association with postmenopausal RPA but found increased risk associated with low and high levels of premenopausal RPA. Huang et al. hypothesized that moderate activity may increase IEOC risk by increasing ovulation in premenopausal women; if true, this could provide support for our observation of increased risk associated with predominantly moderate RPA. The present study's finding of no difference by menopausal status is inconsistent with these results, but due to limitations in sample size it may have been underpowered to detect an association.

A cohort study by Chionh et al. 20 reported nonsignificant increases in ovarian cancer risk for women who reported walking once a week compared with women who reported no walking, a finding that is inconsistent with our statistically significant observation of a 40% lower IEOC risk associated with predominantly mild intensity RPA. However, the study also reported significantly increased risk for those performing “less vigorous” intensity activity one to two times per week (OR = 1.58; 95% CI: 1.01, 2.48) and three or more times per week (OR = 1.62; 95% CI: 1.01, 2.61), comparable with our statistically significant OR of 1.51 for the predominantly moderate intensity group with >540 MET‐min/week. A cohort study by Anderson et al. 21 also reported a positive association for women performing moderate‐intensity RPA more than four times per week, which was not statistically significant (OR = 1.17; 95% CI: 0.78, 1.75). In contrast, six case–control studies 3, 5, 6, 7, 8, 10 and five cohort studies 14, 16, 19, 23, 25 have reported ORs less than 1.0 for moderate intensity RPA, although only two case–control studies 8, 10 and one cohort study 16 were statistically significant.

Our finding of reduced IEOC risk associated with strenuous‐intensity RPA is consistent with five case–control studies 3, 5, 8, 10, 19 and one cohort study 18 which have reported reduced risk associated with vigorous‐intensity RPA, although only two 5, 10 found statistically significant associations. Conversely, seven cohort studies have reported ORs greater than 1.0 14, 16, 19, 20, 21, 23, 25, although only one 21 was statistically significant.

Given this background of inconsistent evidence, a clear‐cut explanation is not forthcoming for our observation of reduced IEOC risk associated with mild‐ and strenuous‐intensity RPA in obese but not nonobese women. Replication will be needed to determine if these are chance findings, especially with the lack of a monotonic trend as indicated by the differences in ORs for moderate RPA compared with the mild and strenuous categories. If the associations observed in the present study prove to be true, then interventions designed to target RPA in obese AA women may be a valuable preventive strategy as the prevalence of obesity is higher among AA women than among white women 37.

This study has several limitations. Although the baseline questionnaire included examples of each exercise intensity, the intensity categories were not mutually exclusive and it is possible that participants reported exercise intensity inaccurately. The self‐reported, retrospective nature of the physical activity data also introduced the possibility of recall bias. To reduce the concern that physical activity habits were influenced by undetected disease, however, the baseline questionnaire asked cases about their physical activity habits 1 year prior to their diagnosis. The lack of more specific information about types of physical activity, which would allow for more precise assignment of MET values, is another limitation. Additionally, the case–control design also introduces the possibility of selection bias. It is possible that controls were healthier and more active than the general population, which could bias effect estimates away from the null; it is also likely that participating cases felt better and were therefore more likely to exercise than those who declined to participate.

Despite these limitations, this study has several strengths. AACES is the largest study of IEOC in AA women to date and this is the first assessment of RPA and IEOC in an exclusively AA population. Another strength is the restriction of referent groups to sedentary participants. Many previous studies have used referent groups combining women reporting low levels of RPA with sedentary participants, which would bias a potential association toward the null 2. Our study is also among the first to evaluate mild intensity as a distinct intensity category in addition to moderate and vigorous.

We found that mild and strenuous RPA may be associated with reduced risk of IEOC. Furthermore, our data suggest that the inverse associations for mild and strenuous RPA may be more pronounced in obese women. Further research is warranted to determine whether these findings can be consistently replicated, with attention to the physiologic basis for the impact of physical activity on ovarian carcinogenesis.

## Conflict of Interest

The authors declare that they have no conflict of interest.
